# Environmentally Relevant Perinatal Lead Exposure Alters Metabolic Pathways Related to Oxidative Stress, Inflammation, and Lipid Metabolism in Mouse Cerebral Cortex and Blood Plasma with Implications for Dementia

**DOI:** 10.1002/alz70860_102954

**Published:** 2025-12-23

**Authors:** Rachel K Morgan, Evelyn K Matei, Tomoko Ishikawa, Junru Pan, Katelyn M Polemi, Dana C Dolinoy, Kelly M. Bakulski, Justin A Colacino

**Affiliations:** ^1^ University of Michigan, Ann Arbor, MI, USA; ^2^ Michigan Alzheimer's Disease Research Center, Ann Arbor, MI, USA

## Abstract

**Background:**

Exposure to neurotoxicants, including lead (Pb), during development are suspected contributors to dementia. Dementia is characterized by neurologic metabolic changes. The metabolic impacts of Pb exposure are not characterized in neurologic or circulating tissues. Here, following perinatal Pb exposure, we evaluated metabolomic profiles in plasma and cerebral cortex from male and female mice at postnatal day 21 (PND21).

**Method:**

Female mice were exposed to 32ppm Pb or control water from two weeks prior to mating through offspring weaning, resulting in human‐relevant blood Pb levels (10ug/dL) in mid‐gestation dams. Plasma and cortex were collected from PND21 offspring. Metabolomics were measured via ultra‐performance liquid chromatography‐mass spectrometry. Two‐way ANOVA identified Pb exposure‐associated biochemicals that displayed significant (*p* <0.05) fold changes (FC), including sex‐stratified analyses (Pb: male cortex *n* = 11, plasma *n* = 10, female cortex *n* = 11, plasma *n* = 9, control: male cortex *n* = 8, plasma *n* = 8, female cortex *n* = 7, plasma *n* = 8).

**Result:**

Pb elevated oxidative stress markers in cortex (methionine sulfoxide FC = 1.16, lanthionine FC = 1.33) and plasma (glycine FC = 1.14, serine FC = 1.24), with stronger effects in females. In the cortex, Pb reduced inflammation markers (FC: 0.57‐0.65), but elevated inflammation markers in plasma (FC: 1.62‐1.97), while Pb reduced kynurenine in both the cortex (FC = 0.77) and plasma (FC = 0.77). Pb exposure also elevated fatty acid metabolites (e.g., medium chain and dicarboxylate fatty acids) in the cortex (FC: 1.18‐1.46) and plasma (FC: 1.36‐2.77), with stronger effects in females.

**Conclusion:**

Pb exposure altered cortex and plasma metabolites related to oxidative stress, inflammation, and lipid metabolism, and the majority of associations were stronger in females. Results suggest metabolites (e.g., kynurenine and fatty acids) may be potential biomarkers of Pb exposure and effects related to neurodegenerative disease. Ongoing work includes analyses in aging mice (18 months old) and comparison to metabolic signatures of dementia.

